# Efficacy and safety of azilsartan medoxomil in the treatment of hypertension: a systematic review and meta-analysis

**DOI:** 10.3389/fcvm.2024.1383217

**Published:** 2024-07-04

**Authors:** Ling Zhu, Guo-Cui Wei, Qing Xiao, Qian-Lan Chen, Qian Zhao, Xiu-xia Li, Ling-ai Pan, Xuan Xiong

**Affiliations:** ^1^ Department of Pharmacy, Sichuan Provincial People’s Hospital, University of Electronic Science and Technology of China, Chengdu, China; ^2^ Department of Critical Care Medicine, Sichuan Provincial People’s Hospital, University of Electronic Science and Technology of China, Chengdu, China

**Keywords:** azilsartan medoxomil, hypertension, diabetes, meta-analysis, angiotensin II receptor blockers (ARBs)

## Abstract

**Background:**

Angiotensin II receptor blockers (ARBs) are utilized for the management of hypertension and diabetes. Previous meta-analyses suggested that azilsartan medoxomil (AZL-M) improved blood pressure (BP) reduction, but there were no safety findings or suggestions for patients with hypertension or diabetes.

**Methods:**

We performed an efficacy and safety meta-analysis of randomized controlled trials (RCTs) evaluating AZL-M therapy for reducing BP in patients with hypertension. Patients with hypertension complicated by diabetes were analyzed. The relevant literature was searched in English and Chinese databases for RCTs involving AZL-M in hypertension. Efficacy variables included the change from baseline in the 24-h mean systolic/diastolic BP measured by ambulatory BP monitoring, the change from baseline in clinic systolic/diastolic BP, and responder rates. Safety variables included total adverse events (AEs), serious AEs, AEs leading to discontinuation, and AEs related to the study drug. The raw data from the included studies were utilized to calculate the odds ratio (OR) for dichotomous data and the mean difference (MD) for continuous data, accompanied by 95% confidence intervals (CIs). Statistical analysis was performed using R software.

**Results:**

A total of 11 RCTs met the inclusion criteria, representing 7,608 patients, 5 of whom had diabetes. Pooled analysis suggested a reduction in BP among patients randomized to 40 mg of AZL-M vs. control therapy [24-h ambulatory blood pressure monitoring (ABPM) mean systolic blood pressure (SBP) (MD: −2.85 mmHg), clinic SBP (MD: −3.48 mmHg), and clinic diastolic blood pressure (DBP) (MD: −1.96 mmHg)] and for 80 mg of AZL-M vs. control therapy [24-h ABPM mean SBP (MD: −3.59 mmHg), 24-h ABPM mean DBP (MD: −2.62 mmHg), clinic SBP (MD: −4.42 mmHg), clinic DBP (MD: −3.09 mmHg), and responder rate (OR: 1.46)]. There was no difference in the reduction of risks, except for dizziness (OR: 1.56) in the 80-mg AZL-M group or urinary tract infection (OR: 1.82) in the 40-mg AZL-M group. Analysis of patients with diabetes revealed that AZL-M can provide superior management, while safety and tolerability were similar to those of control therapy.

**Conclusions:**

AZL-M appears to reduce BP to a greater extent than dose-control therapy and does not increase the risk of adverse events in patients with hypertension and diabetes compared with placebo.

**Systematic Review Registration:**

https://www.crd.york.ac.uk/PROSPERO/display_record.php?RecordID=464284, identifier PROSPERO CRD42023464284.

## 1 Introduction

In the last three decades, despite a stable global age-standardized prevalence, there has been a consistent year-on-year increase in the number of patients diagnosed with hypertension, primarily due to population growth (1). The prevalence of hypertension in China continues to rise due to an aging population. Despite progress, the control rate of hypertension remains low, increasing from 2.8% in 1991 to only 16.8% in 2015. Given the close causal relationship between blood pressure (BP) levels and cardiovascular disease morbidity and mortality, which account for over 40% of all deaths, it is crucial to prioritize blood pressure control (2).

Angiotensin-converting enzyme inhibitors (ACEI) and angiotensin II receptor blockers (ARBs) have been recognized as an effective approach to managing hypertension and are recommended as first-line treatment by various guidelines (3–5). ACEI/ARB agents are particularly recommended for patients with comorbidities such as diabetes (6), heart failure (7, 8), or renal insufficiency (9, 10). Azilsartan medoxomil (AZL-M), the eighth ARB agent approved in China for treating hypertension in 2021, acts as a prodrug that rapidly converts into azilsartan within the body and exhibits a long half-life of approximately 11 h. Based on dose-ranging studies and pharmacokinetic/pharmacodynamic analyses, daily doses of either 40 or 80 mg of AZL-M demonstrate superior efficacy in controlling blood pressure among most patients (11, 12). Previous meta-analyses (13) suggested that AZL-M is more effective in the treatment of hypertension than the other hypertension drugs, but there were no safety findings or suggestions for patients with hypertension and diabetes. To provide clinicians with guidance regarding drug selection and safer usage, we conducted a meta-analysis evaluating both efficacy and safety outcomes from randomized controlled trials (RCTs).

## 2 Methods

### 2.1 Registration of systematic review

This study has been registered in the online platform International Prospective Register of Systematic Reviews (PROSPERO). The protocol of this systematic review and meta-analysis is available in PROSPERO (CRD42023464284). https://www.crd.york.ac.uk/PROSPERO/display_record.php?RecordID=464284.

### 2.2 Search strategy

This study followed the recommendations of the Preferred Reporting Items for Systematic Reviews and Meta-Analysis (PRISMA) protocol (14). The MEDLINE (via PubMed), Embase, Cochrane Library, China National Knowledge Infrastructure (CNKI), WANFANG, and China Biology Medicine disc (CBMdisc) databases were systematically searched from the beginning of the records through 14 September 2023. The search strategy included medical subject heading terms and keywords related to “hypertension,” “high blood pressure,” “azilsartan medoxomil,” and “TAK-491”; two authors independently performed the search. We assessed all relevant English and Chinese articles for eligibility.

### 2.3 Eligibility criteria and data extraction

Studies with the following characteristics were included: (1) adult patients aged >18 years with diagnosed hypertension, with clinic SBP between 150 and 180 mmHg or less; (2) the study design was a prospective randomized controlled clinical trial; and (3) patients were randomly assigned to receive AZL-M vs. any control therapy or placebo.

The exclusion criteria were as follows: (1) non-human studies; (2) non-comparative studies; (3) known secondary hypertension; (4) severe diastolic hypertension (seated DBP at least 114 mmHg); (5) stage IV chronic kidney disease [glomerular filtration rate (GFR) 30 ml/min per 1.73 m^2^]; and (6) type 1 or poorly controlled T2DM (HbA1c < 8%).

Two authors independently reviewed the titles and abstracts to identify potentially relevant studies. The extracted data included study characteristics, patient characteristics, interventions, outcomes, and other relevant findings. A third author cross-checked the extracted data.

### 2.4 Quality assessment and risk of bias

Two independent authors assessed the risk of bias and the quality of all RCTs using the Cochrane Handbook of Systematic Reviews of Interventions (15, 16).

### 2.5 Outcomes and statistical analysis

The primary outcome measures included the change from baseline in the 24-h mean systolic blood pressure (SBP) measured by ambulatory blood pressure monitoring (ABPM) (24-h ABPM mean SBP), change from baseline in clinic SBP, responder rates (RRs), total adverse events (AEs), serious AEs, AEs leading to discontinuation, and AEs related to the study drug. Secondary outcomes included the change from baseline in the 24-h mean diastolic blood pressure (DBP) measured by ambulatory blood pressure monitoring (24-h ABPM mean DBP), change from baseline in clinic DBP, and adverse events such as headache, dizziness, hyperlipidemia, urinary tract infection, hypotension, and nasopharyngitis.

AZL-M (40 or 80 mg) was chosen as the comparator for control therapy in this meta-analysis. Statistical analysis was performed using R software 4.3. The raw data from the included studies were utilized to calculate the odds ratio (OR) for dichotomous data and the mean difference (MD) for continuous data, accompanied by 95% confidence intervals (CIs). These measures were pooled using a random-effects model. The findings of the pooled studies were presented through forest plots. Egger's (17) test and funnel plots were employed to assess publication bias for effectiveness outcomes and adverse events. Heterogeneity was evaluated and categorized as low (<25%), moderate (25%–75%), or high (>75%) using Higgin's *I*^2^ tests. A *P*-value of 0.05 was considered significant for all analyses.

## 3 Results

### 3.1 Baseline characteristics

**Figure 1:**
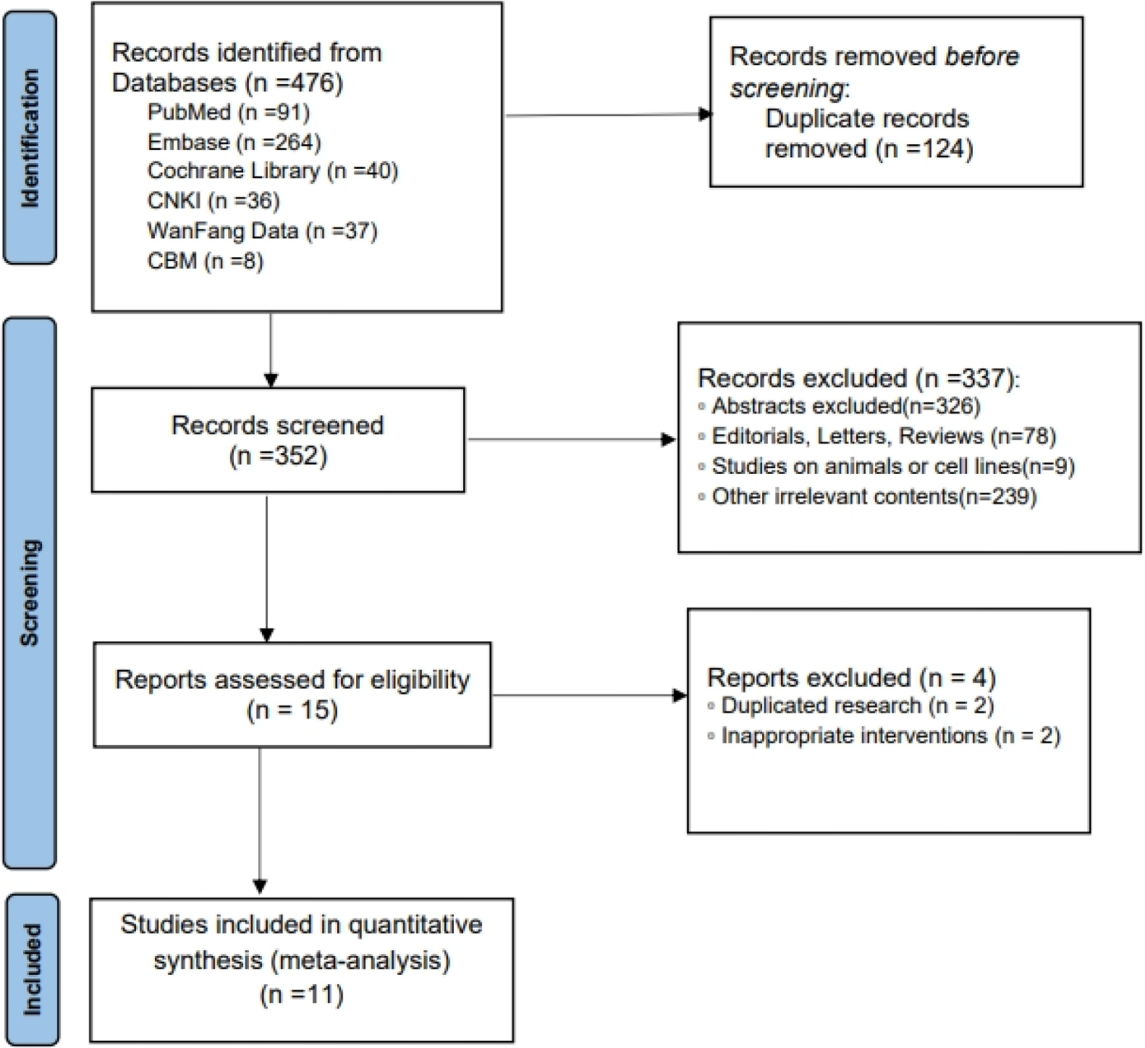
PRISMA diagram of the systematic review search strategy.

**Figure 2:**
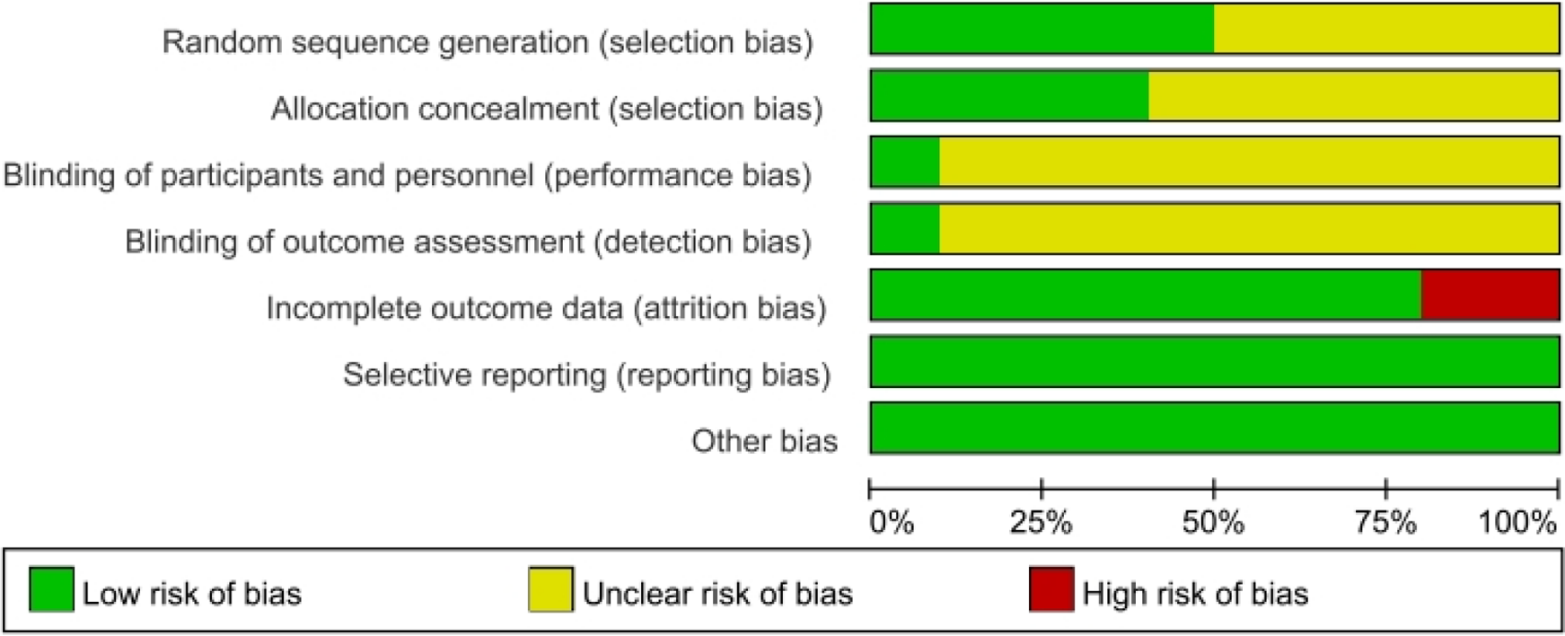
Methodological quality graph: author's judgments about each methodological quality item are presented as a percentage across all included studies.

A total of 11 RCTs (18–28) met the inclusion criteria, representing 7,608 patients (Figure 1). The quality assessment for the included studies is presented in Figure 2. Among the included trials, six were ARB-controlled trials (18, 20, 21, 25–27) (olmesartan, telmisartan, valsartan), two were ACEI-controlled trials (24, 28) (ramipril, benazepril), one amlodipine plus placebo-controlled trial (19), and four were placebo trials (18, 21–23). Almost all the studies included intervention groups with 40 and 80 mg doses of AZL-M, while one study had two different ARB control therapies. Follow-up ranged from 6 to 24 weeks. Despite the noted heterogeneity in design between the trials, there was sufficient similarity between the populations and the hypotheses to merit the inclusion of all 11 trials in the quantitative meta-analysis. Except for Peng et al. (28), which had a population of hypertension and heart failure, they all have the same population of hypertension (Table 1).

**Table 1:** Baseline characteristics of the study participants.

					Treatment				Control			
First author (year)	Country	Populations	Study duration (weeks)	Total sample	Dose of the drug (mg/day)	Patients in AZL-M	Age, mean ± SD	Male (%)	Dose of the drug (mg/day)	Patients in control	Age, mean ± SD	Male (%)
Bakris (2011) (25)	United States, Peru, Argentina, Mexico	Primary hypertension	6	1,275	AZL-M 20 mg	283	57.1 ± 11.0	133 (47.0)	Olmesartan-M 40 mg	282	58.9 ± 11.6	140 (49.6)
					AZL-M 40 mg	283	57.4 ± 9.6	142 (50.2)	Placebo	142	59.4 ± 10.5	76 (53.5)
					AZL-M 80 mg	285	58.1 ± 11.6	149 (52.3)				
Bönner (2013) (24)	Europe, Russia	Stage 1 or 2 hypertension	24	884	AZL-M 40 mg	295	56.0 ± 11.5	159 (53.9)	Ramipril 10 mg	295	56.6 ± 10.5	146 (49.5)
					AZL-M 80 mg	294	56.8 ± 11.3	158 (53.7)				
Garg (2020) (27)	India	Primary hypertension	12	700	AZL-M 81 mg	350	50.6 ± 15.0	196 (56.0)	Telmisartan 40 mg	350	49.6 ± 13.6	203 (58.0)
Johnson (2017) (23)	United States (African-American)	Stages 1 or 2 systolic hypertension	6	413	AZL-M 82 mg	138	52.0 ± 11.0	60 (44.0)	Placebo	138	52.0 ± 11.0	60 (44.0)
					AZL-M 83 mg	137	51.0 ± 10.0	58 (42.0)				
Juhasz 2018) (22)	Korea	Essential hypertension	6	328	AZL-M 84 mg	132	59.8 ± 10.8	95 (72.0)	Placebo	65	58.8 ± 10.2	51 (78.5)
					AZL-M 85 mg	131	58.3 ± 11.6	93 (71.5)				
Perez (2017) (21)	United States, Mexico, Argentina, Peru	Essential hypertension	8	449	AZL-M 86 mg	65	54.0 ± 10.0	36 (55.0)	Olmesartan-M 20 mg	63	53.4 ± 11.0	29 (63.0)
					AZL-M 87 mg	63	56.5 ± 8.5	31 (49.0)	Placebo	61	56.0 ± 11.4	29 (46.0)
					AZL-M 88 mg	64	54.6 ± 9.1	34 (53.0)				
					AZL-M 89 mg	62	55.3 ± 9.8	29 (47.0)				
					AZL-M 90 mg	64	53.5 ± 11.0	36 (56.0)				
Weber (2014) (19)	United States, Peru, Mexico, and Chile	Stage 2 hypertension	6	565	AZL-M 91 mg	189	58.0 ± 11.0	91 (48.0)	Placebo + amlodipine 5 mg	189	59.0 ± 11.0	94(50.0)
					AZL-M 92 mg	188	58.0 ± 12.0	103 (55.0)				
White (2011) (18)	Guatemala, Mexico, Peru, Puerto Rico, United States	Stage 1 or 2 hypertension	6	1,291	AZL-M 93 mg	280	57.0 ± 12.0	148 (53.0)	Valsartan 320 mg	282	56.0 ± 11.0	152 (54.0)
					AZL-M 94 mg	285	56.0 ± 11.0	151 (53.0)	Olmesartan-M 40 mg	290	56.0 ± 11.0	161 (55.0)
									Placebo	154	56.0 ± 11.0	89(58.0)
Sica (2011) (20)	United States, Peru, Argentina, Mexico	Stage 1 or 2 hypertension	24	984	AZL-M 40 mg	327	57.8 ± 12.1	164 (50.2)	Valsartan 320 mg	328	58.1 ± 10.9	176 (53.7)
					AZL-M 80 mg	329	56.8 ± 10.7	169 (51.4)				
Wu (2020) (26)	China	Essential hypertension	8	612	AZL-M 40 mg	199	57.4 ± 9.5	107 (65.8)	Valsartan 160 mg	204	56.8 ± 9.5	130 (63.7)
					AZL-M 80 mg	209	57.0 ± 9.9	115 (55.0)				
Tao (2023) (28)	China	Hypertension and heart failure	8	107	AZL-M 80 mg	54	53.8 ± 8.7	28 (52)	Benazepril 10 mg	53	54.3 ± 9.2	29 (55)

### 3.2 Efficacy meta-analysis

**Figure 3:**
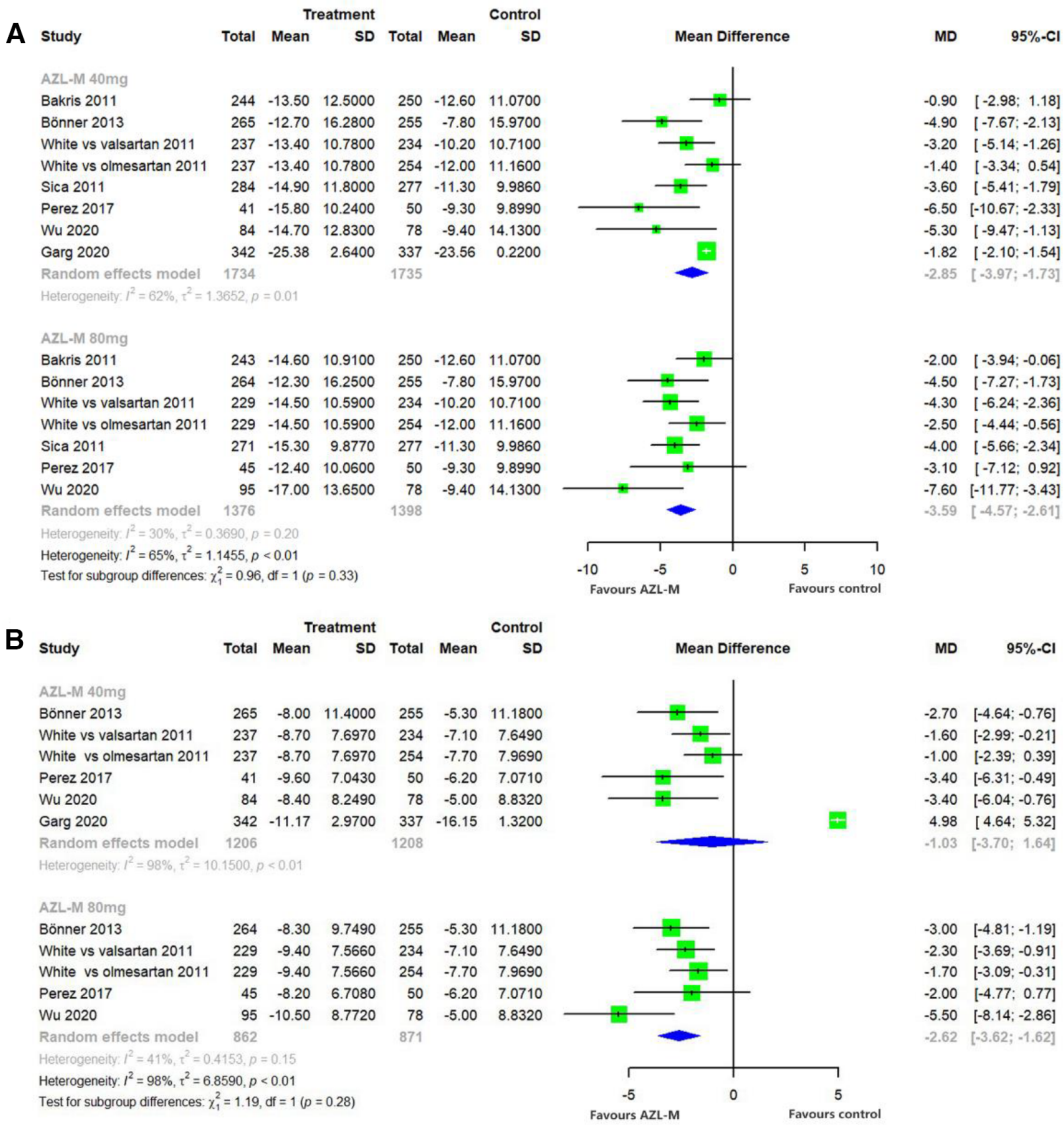
Forest plot of 24-h ABPM mean SBP (**A**) and 24-h ABPM mean DBP (**B**) among hypertensive patients randomized to azilsartan medoxomil vs. control therapy.

Changes from baseline in 24-h ABPM mean SBP were significantly greater with 40 mg of AZL-M (MD: −2.85 mmHg, 95% CI: −3.97 to −1.73 mmHg, *p *< 0.05) and 80 mg of AZL-M (MD: −3.59 mmHg, 95% CI: −4.57 to −2.61 mmHg, *p *< 0.05) than with control therapy. When compared with 24-h ABPM mean DBP, there was a statistically significant difference in the 80-mg AZL-M group (MD: −2.62 mmHg, 95% CI: −3.62 to −1.62 mmHg, *p *< 0.05), whereas 40 mg of AZL-M was non-inferior to control therapy (MD: −1.03 mmHg, 95% CI: −3.70 to 1.64 mmHg, *p *= 0.57) (Figure 3).

**Figure 4:**
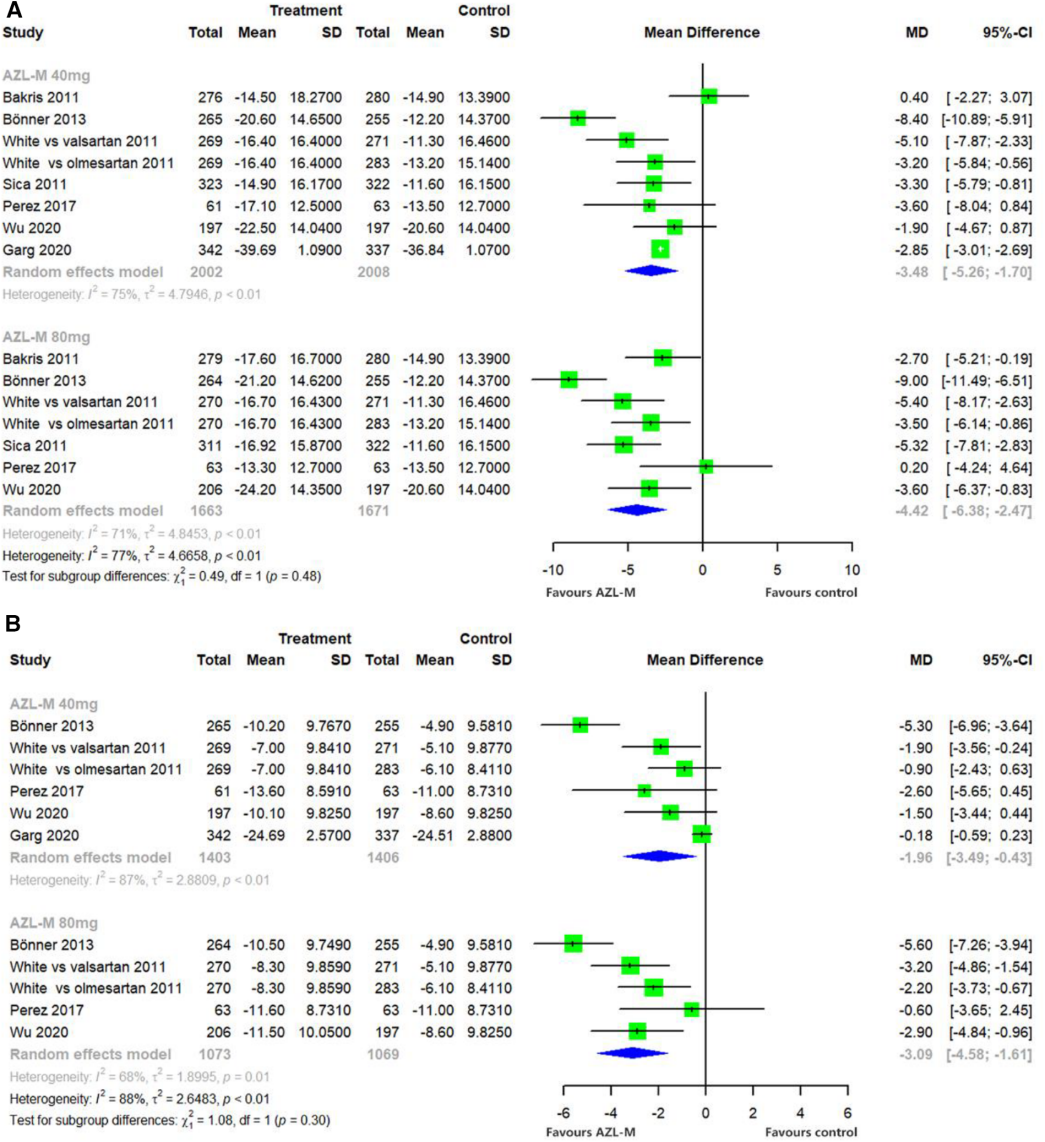
Forest plot of clinic SBP (**A**) and clinic DBP (**B**) among hypertensive patients randomized to azilsartan medoxomil vs. control therapy.

Changes from baseline in the clinic SBP compared with control therapy demonstrated a statistically significant difference in the 40-mg AZL-M group (MD: −3.48 mmHg, 95% CI: −5.26 to −1.70 mmHg, *p *< 0.05) and the 80-mg AZL-M group (MD: −4.42 mmHg, 95% CI: −6.38 to −2.47 mmHg, *p *< 0.05). In contrast, the clinic DBP also showed a statistically significant difference in the 40-mg AZL-M group (MD: −1.96 mmHg, 95% CI: −3.49 to −0.43 mmHg, *p *< 0.05) and the 80-mg AZL-M group (MD: −3.09 mmHg, 95% CI: −4.58 to −1.61 mmHg, *p *< 0.05) compared to the control therapy (Figure 4).

**Figure 5:**
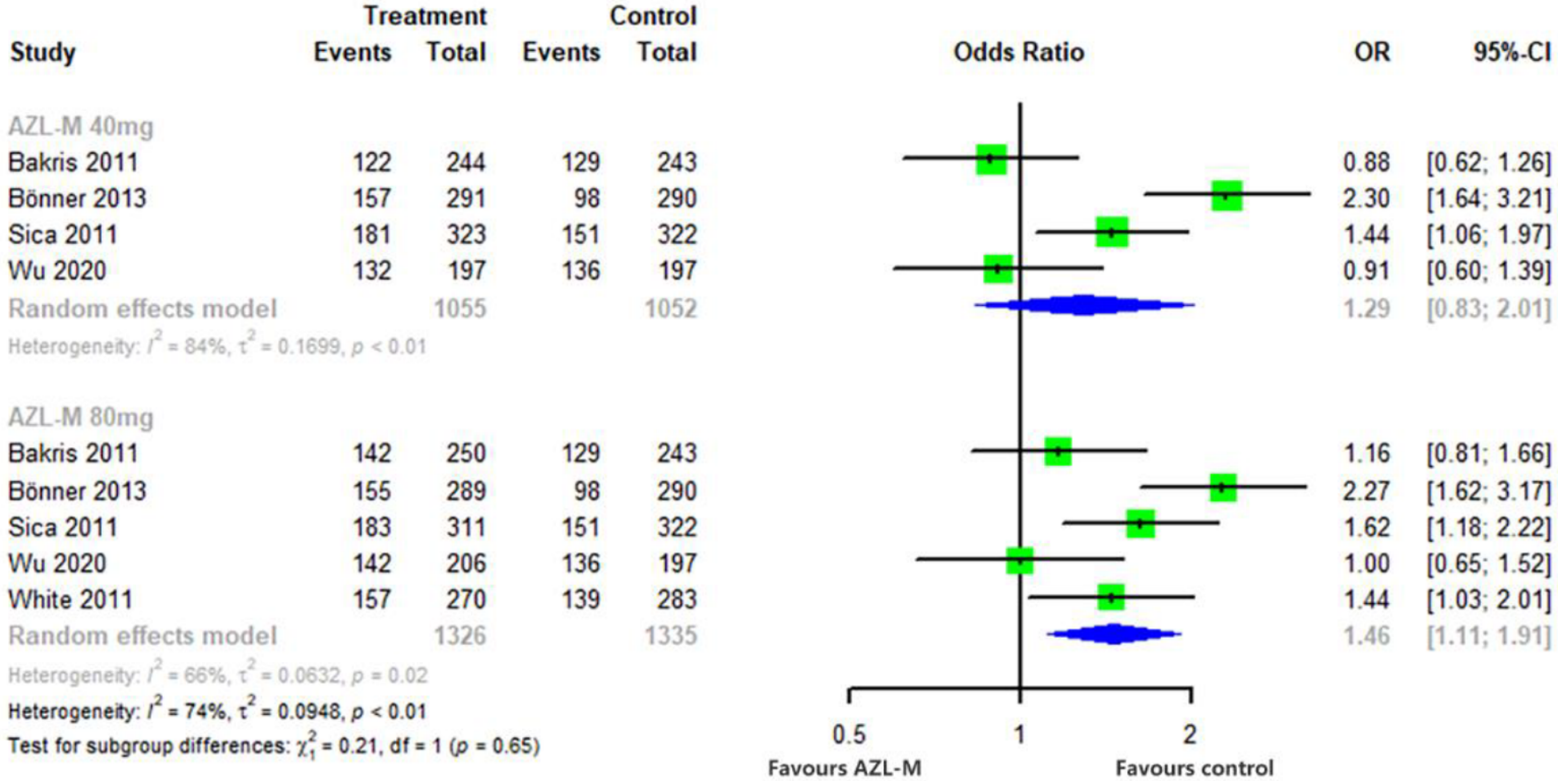
Forest plot of responder rates among hypertensive patients randomized to azilsartan medoxomil vs. control therapy.

The proportion of patients who achieved a reduction of clinic SBP to <140 mmHg or a reduction of >20 mmHg was significantly higher in the 80-mg AZL-M group (OR: 1.46, 95% CI: 1.11–1.91, *p *= 0.256) compared with control therapy. Similarly, 40 mg of AZL-M was non-inferior to control therapy (OR: 1.29, 95% CI: 0.83–2.01, *p *< 0.05) (Figure 5).

### 3.3 Safety meta-analysis

In the safety analysis set, all the pooled data were compered in two groups, namely, control therapy and placebo, if available. The safety meta-analysis is presented in Table 2. The results revealed that there was no difference in the reduction of risks for total adverse events, AEs leading to discontinuation, serious AEs, and AEs related to the study drug. However, there was a higher risk of dizziness (OR: 1.56, 95% CI: 1.08–2.26, *p *< 0.05) in the 80-mg AZL-M group and more risks of urinary tract infection (OR: 1.82, 95% CI: 1.14–2.90, *p *< 0.05) in the 40-mg AZL-M group. Nevertheless, there was no difference in the risk of headache, hyperlipidemia, hypotension, or nasopharyngitis.

**Table 2:** Results of the safety meta-analysis of azilsartan medoxomil vs. control therapy and placebo.

Adverse events	AZL-M vs. control						AZL-M vs. placebo					
	40 mg			80 mg			40 mg			80 mg		
	OR (95% CI)	*P* ^a^	*I*^2^ (%)	OR (95% CI)	*P* ^a^	*I*^2^ (%)	OR (95% CI)	*P* ^a^	*I*^2^ (%)	OR (95% CI)	*P* ^a^	*I*^2^ (%)
Total adverse events	0.96 (0.84–1.08)	0.48	0	1.14 (1.00–1.31)	0.05	0	0.98 (0.80–1.19)	0.83	0	0.99 (0.82–1.21)	0.93	0
Serious AEs	0.77 (0.45–1.33)	0.35	0	1.03 (0.62–1.70)	0.92	0	0.75 (0.29–1.94)	0.55	17	0.82 (0.33–2.03)	0.67	0
AEs leading to discontinuation	0.90 (0.63–1.30)	0.59	0	1.20 (0.83–1.73)	0.33	0	0.78 (0.40–1.52)	0.47	1	0.88 (0.46–1.69)	0.70	0
AEs related to the study drug	1.03 (0.70–1.51)	0.90	5	1.07 (0.73–1.56)	0.74	0	—	—	—	—	—	—
Headache	0.87 (0.67–1.12)	0.30	0	0.79 (0.59–1.05)	0.11	28	0.82 (0.53–1.26)	0.39	0	0.87 (0.57–1.32)	0.54	24
Dizziness	1.32 (0.93–1.89)	0.12	0	1.56 (1.08–2.26)	<0.05	0	1.17 (0.62–2.21)	0.63	0	1.27 (0.69–2.39)	0.45	0
Urinary tract infection	1.82 (1.14–2.90)	<0.05	0	1.53 (0.95–2.48)	0.08	0	0.75 (0.32–1.70)	0.51	0	0.57 (0.23–1.42)	0.23	0
Hyperlipidemia	0.98 (0.55–1.72)	0.93	0	1.14 (0.67–1.97)	0.62	27	—	—	—	—	—	—
Nasopharyngitis	0.83 (0.49–1.41)	0.50	0	0.67 (0.39_1.17)	0.16	27	—	—	—	—	—	—
Hypotension	3.83 (0.94–15.53)	0.06	0	2.22 (0.50–9.97)	0.29	0	—	—	—	—	—	—

^a^ Text for the subgroup effect.

### 3.4 Hypertension with diabetes

We conducted an analysis on patients with hypertension combined with diabetes. Among the included studies, five (18, 20, 22, 23, 25) involved patients with diabetes. However, studies by Johnson et al. (23) and Juhasz et al. (22) were compared to a placebo, and comparable data from the others was unavailable. Nevertheless, one article (29) just included outcomes from the three RCTs (18, 20, 25), comparing the effects of AZL-M with olmesartan and valsartan on ambulatory and clinic blood pressure in patients with type 2 diabetes and prediabetes. The analyses indicate that AZL-M at the approved dose of 80 mg provides superior management, with safety and tolerability similar to the control therapy (29).

### 3.5 Publication bias and sensitivity analysis

**Figure 6:**
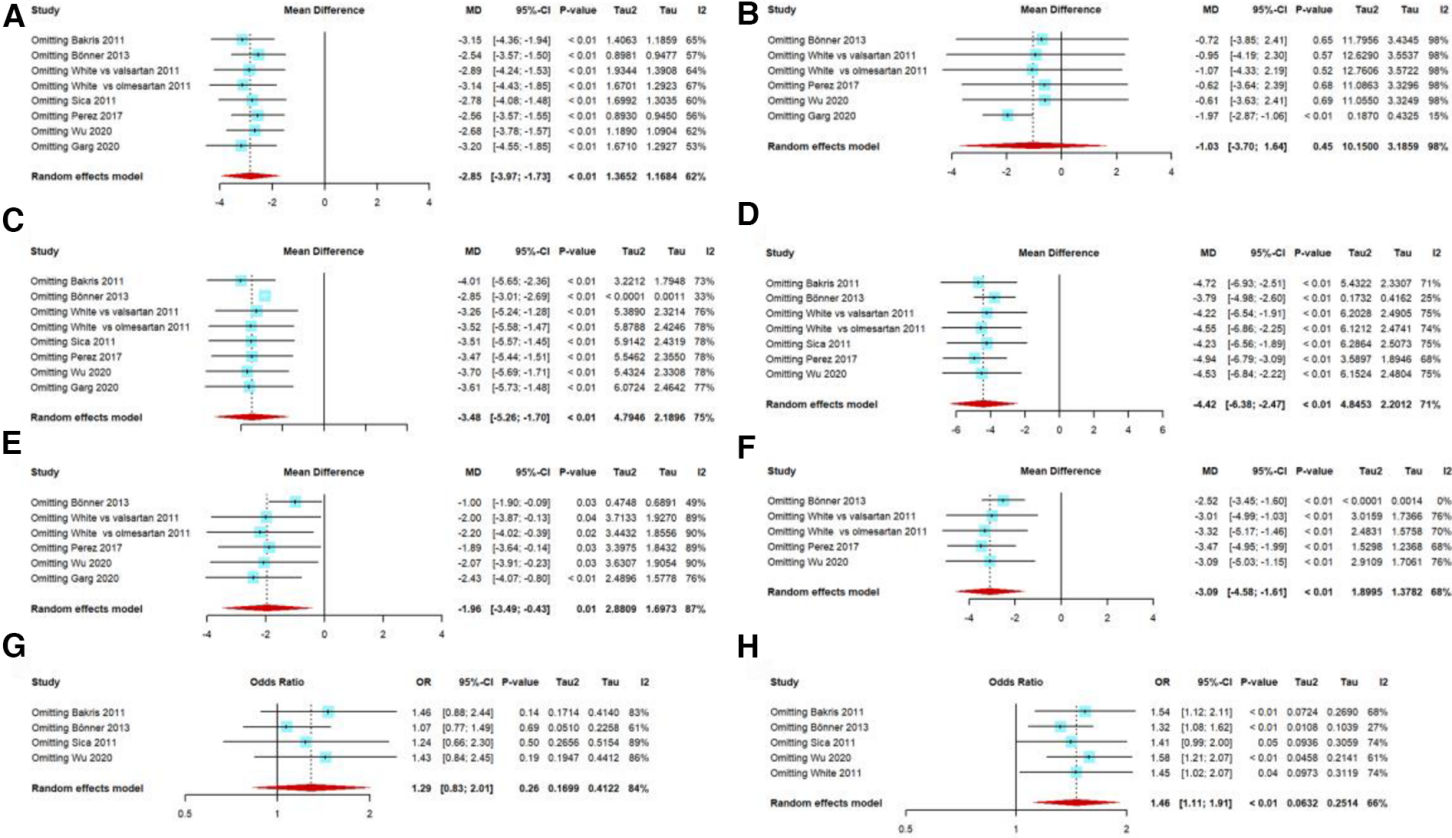
Sensitivity analysis: 40 mg AZL-M vs. control therapy in 24-h ABPM mean SBP (**A**)/DBP (**B**), 40 mg AZL-M vs. control therapy in clinic SBP (**C**)/DBP (**E**), 80 mg AZL-M vs. control therapy in clinic SBP (**D**)/DBP (**F**), and responder rate on 40 mg (**G**)/80 mg (**H**) AZL-M vs. control therapy.

Publication bias tests were performed with >10 studies according to the guidelines, but our included studies were fewer than 10. The outcomes of the efficacy analyses had several heterogeneous results. We performed several sensitivity analyses, and excluding any single trial from the analysis did not substantially alter the overall results, except for 40 mg of AZL-M for 24-h ABPM mean DBP; when we excluded the trial by Garg et al. (27), it showed a statistically significant result favoring 40-mg AZL-M therapy (MD: −1.97 mmHg, 95% CI: −2.87 to −1.06 mmHg, *p* < 0.01) (Figure 6).

## 4 Discussion

We conducted a meta-analysis on a randomized controlled trial of 40 and 80 mg of AZL-M, which are approved dosages for hypertension treatment in China. The analysis compared these dosages with control therapy and placebo, revealing that AZL-M demonstrated superior reductions in mean SBP and DBP measured by 24-h ABPM, as well as clinic SBP, clinic DBP, and responder rate. These efficacy results are consistent with previous research (13) and remained robust in sensitivity analyses except for the study by Garg et al. (27), which impacted the overall outcome. We attribute this to differences in patient selection criteria and blinding methods between Garg et al. and other studies. The study by Garg et al. included a patient with a clinic SBP of ≥150 to ≤180 mmHg (stage 2), while the other studies included stage 1 patients. The study by Garg et al. was an open-label, assessor-blinded trial, which introduced systematic bias because investigators or trial participants were aware of the treatment assignment.

ARBs are typically well tolerated (30), and the side effect profile is generally similar to that seen with ACE inhibitors, although hypotensive symptoms appear to be more common with ARBs (31). The most commonly reported adverse events in AZL-M include headache, dyslipidemia, dizziness, and hyperlipidemia. The incidence of hypotension appears to be low, but there is a higher incidence of dizziness and a lower incidence of urinary tract infection based on this analysis. The pooled studies had varying durations ranging from 6 to 24 weeks; however, longer follow-up studies have indicated similar results. The observational study by Gitt et al. (32) showed improvements in BP control, while the study by Bakris et al*.* (33) demonstrated tolerable profiles over 52 weeks.

The efficacy analysis consisted of 24-h mean ABPM SBP/DBP and clinic SBP/DBP. Blood pressure measured by ABPM can differentiate between white-coat hypertension and masked hypertension (34)and can predict all-cause mortality and cardiovascular events (35). Patients with hypertension can benefit from treatment with AZL-M in reducing cardiovascular events (28, 36). Hypertension increases the risk for a variety of cardiovascular diseases (37); for each 20/10 mmHg increase in systolic/diastolic blood pressure, there is a doubling of coronary heart- and stroke-related mortality (38, 39).

AZL-M is a prodrug that is rapidly hydrolyzed to the active moiety, azilsartan, with a half-life of approximately 11 h. Azilsartan inhibits angiotensin II's vasoconstrictor and aldosterone-secreting effects by selectively blocking the binding of angiotensin II to the AT_1_ receptor in vascular smooth muscle and adrenal gland tissues (azilsartan has a stronger affinity for the AT_1_ receptor than the AT_2_ receptor) (40). The action is independent of the angiotensin II synthesis pathways. Beyond BP control, azilsartan has potential effects that include amelioration of the deleterious effects of angiotensin II such as cardiac hypertrophy, fibrosis, insulin resistance, and stabilization of coronary plaques (41); as also, it causes positive changes in leptin, C-reactive protein, IL-6, adiponectin levels (42). In healthy individuals, no AZL-M dose adjustments are required based on age, sex, or race (black/white) (43).

Furthermore, ARBs are extensively utilized for the management of hypertension, chronic kidney disease, heart failure, and diabetes. We analyzed the data of patients with hypertension and diabetes; one article compared the effects of AZL-M with olmesartan and valsartan and indicated that 80 mg of AZL-M provides superior management. Fixed-dose combinations of AZL-M and chlorthalidone have shown significant reductions in systolic blood pressure along with good tolerability among hypertensive participants with stage 3 chronic kidney disease (33). In patients with heart failure with preserved ejection fraction (HFpEF), azilsartan improved the diastolic function parameters of the left ventricle (44). In patients with hypertension who are overweight or obese, AZL-M also provided good BP control (45).

However, our analysis has several limitations. First, considerable heterogeneity was observed in the results of the efficacy meta-analysis, which may be attributed to factors such as race, treatment duration, and study methodologies. Second, because the duration of treatment was relatively short whereas hypertension requires lifelong management, this study could not adequately capture long-term benefits or side effects. Third, we relied on data from randomized controlled trials where enrolled patients may not represent those typically encountered in clinical practice. Hypertension is often accompanied by multiple complications, yet we included only one study related to heart failure.

## 5 Conclusion

In conclusion, AZL-M appears to provide a greater reduction in BP than control therapy in patients with hypertension and has no greater risk of adverse events than control therapy or placebo in patients with hypertension and diabetes. Nonetheless, more evidence is still needed.

## Data Availability

The original contributions presented in the study are included in the article/Supplementary Material; further inquiries can be directed to the corresponding authors.
